# A Plea for Co-Operation between the War Office and the National Insurance Commissioners

**Published:** 1915-05-01

**Authors:** 


### MEDICAL TREATMENT OF SOLDIERS ON SICK LEAVE.

### A Plea for Co-operation Between the War Office and the National Insurance Commissioners.

There never was a time when the hearty co-operation between the various Government departments was more needed than it is to-day. The war has created new conditions in every sphere of life, and has shown in multifarious ways the mutual dependence of every section of the community upon every other section. These new conditions have necessitated departures from accepted conventions in social as well as political life, and altogether it may be stated that we are living in a new era, when barriers, supposed to be insuperable, have been broken down, and new relationships, considered impossible, have been set up in almost every conceivable branch of our national existence. It is only necessary to point to the emergency measures that have been taken by the Government to meet the pressing and altogether exceptional and undreamt-of circumstances in which we are living, and through which we have recently passed, to make it at once apparent that special conditions require special treatment.

During the past few weeks in particular it has become increasingly evident that the organisation of the War Office, magnificent though it has been, and in spite of its successful operations in raising, training, and equipping the new Armies, is in danger of being overloaded by a mass of detail work which might very well be done by other Government departments. A commencement has already been made for relieving the War Office in regard to the provision of munitions for the Armies at the Front, and for those so shortly to be sent abroad, but there is at least one other direction in which co-operation between the War Office and another Government department calls for immediate attention.

### The Need for Medical Treatment.

We refer to the question of the provision of medical treatment for soldiers on sick leave, and suggest that extended use should be made of the organisation which has been set up, at very considerable expense to the nation, and with mature thought and attention to detail, for the provision of medical benefit under the National Insurance Acts. We have already pointed out in former issues that men who were insured under the National Insurance Acts, and who have joined the Forces, are treated in regard to their insurance as members of the Regular Forces. That is to say, they pay a smaller weekly contribution than they did in civil employment, and at the same time forego their right to sickness and medical benefits until they are discharged. While serving with the Army it is supposed that neither of these benefits is required, for when the soldier is incapacitated for his ordinary duties by illness he is maintained in barracks or hospital as though he were well, and his pay goes on just the same. Moreover, medical attendance and treatment is provided, and in ordinary circumstances the man is under the immediate charge of his regimental doctor at headquarters. These normal conditions do not now, however, prevail. It is true that every care is expended in regard to the health of the troops both while they are in training and while serving at the Front, but when a soldier has become incapacitated during training, or has been invalided home from the Front, he is frequently discharged from hospital long before he has fully recovered, in order to make room for more serious and urgent cases. He is sent to his own home convalescent, but still needing medical care and attendance to restore him to that full health and vigour which is necessary if he is once again to become an effective unit in the Army, or to fit him to take his accustomed place in civil life. His home may be, and most frequently is, many miles—perhaps hundreds of miles—from his regimental headquarters, and it is impossible for him to secure the advantage of the medical treatment of the regimental doctor. He may also be living far away from all hospital facilities, and in these circumstances what is more natural than to turn for the required medical attendance to the doctor who was responsible for his treatment in normal times under the National Insurance Acts? Unfortunately for him, as he has not received his discharge from the Army, he finds upon calling upon his panel doctor that the doctor has received specific instructions from the Insurance Commissioners that if he treats a disabled soldier who has not obtained his discharge he is not entitled to treat him as a panel patient. Any treatment given by the doctor in these circumstances is chargeable to the soldier on sick leave just as though he were a private patient, in spite of the fact that before joining the Army he could have obtained such treatment, free of charge, as a benefit under the National Insurance Acts. With the reduced means available—as they frequently are, the Army pay being less than the man's normal earnings—it is little wonder that the man feels that he has a grievance. It is always desirable to remove every possible cause of complaint, but this is not the main reason for our advocacy for immediate action. That reason is, that unless action is taken the fact that medical treatment has to be paid for out of private—and often meagre—resources will act as a deterrent to the men in seeking such treatment, and will thus prejudice the rapid recovery which is so essential.

### Immediate Action Necessary.

The subject has been brought prominently to the notice of the authorities by correspondence in the public Press and in other ways. We, ourselves, commented upon the anomalies of the situation several weeks ago, but although two amending Acts have already been placed on the Statute Book to meet certain problems in connection with National Insurance raised by the war, nothing has apparently yet been done in the important matter to which we now draw special attention. The extent of the correspondence in the Press leaves it beyond doubt that the matter is one of urgency, and in order to meet it various suggestions have been put forward—such, for instance, as making use of the Soldiers' and Sailors' Help Society. We are convinced, however, that nothing short of complete co-operation between the War Office and the Insurance Commissioners, whereby the organisation already in existence under the National Insurance Acts can be utilised to the full, will meet the circumstances to the extent that is desirable.

It is sure to be stated that the Insurance Commissioners are bound by the Acts, and have no power to provide the medical treatment for the soldiers on sick leave, nor the funds from which such treatment could be furnished. The answer to this is that if the Acts are so restricted they must be extended in an appropriate manner to meet the necessity of the situation. We are not so sure, however, that any such legislation is required, or that it is necessary that the cost of the medical treatment should be a charge on the Insurance funds. It should surely be possible for each soldier to be furnished with a voucher, when he is sent home on sick leave, entitling him to receive medical treatment from his panel doctor. The charge for this treatment would remain against the War Office, to be provided out of Parliamentary funds, and it would only necessitate a simple process of accounting, with the aid of the Insurance Committees and the other organisations already in existence under the National Insurance Acts, to make the system work effectively. As an instance of what can be done by co-operation and agreement, without the sanction of legislative enactment, the recent action taken by the representatives of the largest organisations among the approved societies to prevent the altogether unnecessary expense and trouble involved by the transference of members from one society to another, may be cited as an illuminating example.

If it is possible for societies, which at the inception of the National Insurance scheme were antagonistic to each other, to arrive at such an agreement merely for the sake of saving of trouble and expense, we have surely a right to expect that such a vital matter as the provision of medical treatment for the soldiers who have been incapacitated in the service of the country should not be rendered impossible for lack of co-operation between two Government departments.

